# New Perspectives on Specific Immune-Depletion Technique Using Monoclonal Antibodies against Small Active Molecules in Herbs

**DOI:** 10.1155/2014/393680

**Published:** 2014-03-17

**Authors:** Xue-Qian Wang, Fa-Feng Cheng, Hui-Hua Qu, Yan Zhao, Cai Yu, Yuan-Jun Liu, Wen-Xiang Zhu, Qing-Guo Wang

**Affiliations:** ^1^College of Basic Medicine, Beijing University of Chinese Medicine, Beijing 100029, China; ^2^“The Basic Research of Classic Recipe Application” Innovation Research Team, Beijing University of Chinese Medicine, Beijing 100029, China; ^3^Scientific Research Center, Beijing University of Chinese Medicine, Beijing 100029, China

## Abstract

One of the main focuses in Chinese Medicine research is the identification of efficacious components in Chinese herbal medicine (CHM). Studies in such area are difficult due to the complexity and the synergistic characteristics of CHM. Current methods to track and separate active components are not adequate to meet the needs of revealing effects and identify substances and pharmacological mechanisms, which directly restrict the modernization and globalization of CHM. In this paper, a new methodology to deplete a single active component via immunoassay was introduced. The specific active component in a CHM mixture can then be identified and studied through comparative analyses of the pharmacological effects before and after immune depletion. With this new methodology, degree of contribution of a particular component to the whole complex herbal mixture can be elucidated, and its synergistic property with other components can be determined. The new method can reflect not only the overall combined pharmacological effects of CHM but also the effect of individual component. It is an effective way to explain the degree of contribution of one specific component to the overall activity of a CHM prescription.

## 1. Introduction

Complexities are the basic character of Chinese Herbal Medicine (CHM, Zhong Yao in Chinese). The composition of CHM is complex in terms of its chemical structure and therapeutic effects. The pharmacodynamics of CHM is generated from the combined effects of different herbal components in a complex CHM mixture. And such combination is a result of a linear or nonlinear accumulation of synergistic or antagonistic medicinal interactions in vivo. In order to properly and comprehensively study and understand the pharmacological mechanism and synergistic effects in CHM and to establish a standard CHM quality control (QC), it is vital to identify the most active component in any given CHM prescription, the “principal component” (PC). And how to accurately explain the relationship between the therapeutic effects of a CHM preparation and the variation in concentration of principal compound is one of the key questions that needed answers in CHM research. This will be a key to develop CHM with factual bases.

When it comes to establishing connections between a principal component and a primary medicinal property of a herb, simply focusing on one specific chemical compound is insufficient. The pharmacological action of a herb is far more sophisticated and every active component should be taken into consideration and analysed as a whole. Even though the dominant component may be the major contributor to the herb's pharmacological function, but its synergistic relationship with other components is just as important. Current existing methods are unsubstantial to satisfy the needs of material-based CHM research. New ideas and perspectives are needed to explore new techniques and applications.

The primary objective in the CHM research is to identify the principal component and determine its main contributions to the pharmacodynamics of component herbs. In recent years, with the advancements in detection method development and the application of “fingerprint” technology, researchers have successfully isolated and analysed the primary active components in herbs and herbal mixtures, but study on CHM trace elements has just begun. It was necessary to study herbal medicine in vivo and define the absorption, metabolism, and distribution in different organs, tissues and cells, but to reveal the whole medicinal component system was not easy. Moreover, after the absorption and metabolism, it was exceedingly difficult to identify the pharmacological mechanism in vivo because of the content descend and conformational change of active components. Due to the complexity of CHM, it has been difficult to examine its pharmacodynamics and pharmacokinetics in vivo. However, scholars in the field of CHM research continuously come up with better strategies and have made some breakthrough in the area [1, 2]. One practical and effective research strategy currently adopted in the field is using monomeric component as a starting point for quality control, pharmacokinetic analysis, and drug innovation [3].

## 2. Current Status on Material-Based CHM Research

Progress has been made in principal component identification [4–7], which leads to making great strides in both of pharmacodynamics and pharmacokinetics determinations in CHM research. Advancements have been made in correlating the pharmacological properties of primary active components in certain CHM prescriptions and their consistency of therapeutic effects [8]. Some scholars have introduced the best ratio of principal components in some CHM decoction and documented the influences of different decocting methods. These new research updates provide foundations for new experiments and assist in the establishment of standard CHM QC systems [9]. The degree of contribution of principal components in herbal medicine is still a high-profile and also a difficult topic in the CHM research field. Many questions have been raised. Can an identified principal component truly represent the therapeutic effect of a CHM prescription? What are the real-world applications of such principal component? In a complex CHM mixture, which component can serve as the standard QC? And how can altering the level of this component influence the efficacy of CHM? Much work is still needed to be done in the future.

The unique nature of CHM is created from the combined actions of different components. In order to achieve standardized QC, establish material-based pharmacodynamics, and analyse the metabolism of any of the CHM prescriptions, it is necessary to correlate the medicinal property of the principal component identified and the prescription's known therapeutic effects. And it is also necessary to correlate the amount of principal component present and the overall CHM therapeutic performance. A “quantity-effect” (QE) relationship of the principal component can then be generated and studied. This QE relationship can then be further applied into other different research aspects, such as the metabolism, the in vivo distribution, and the medicinal behaviors of CHM. Then, the accurate functional consistency of herbs and the main active component will be described. How to accurately and precisely establish such relationship is a critical issue being faced in basic CHM research. Such relationship can also affect the planting, harvesting, processing, and quality control processes of CHM. It is also a significant foundation for future CHM researches in areas such as herbal compatibility, CHM pharmacokinetics, and CHM pharmacodynamics, which can ultimately lead to a more effective, more stable, and safer application of CHM principal component.

## 3. Limitations of Current Research Methods

Defining the corresponding mechanisms of CHMs is enormously challenging due to the complexity and unique nature of each herb. A single herb possesses a complex system that consists of a broad spectrum of compounds. When a herb is introduced into an animal or a human, each of the compound's pharmacodynamic characteristics will be altered after they are metabolized or interacted with other compounds, producing a different therapeutic effect. The interactions between various components in herbal medicine can be synergistic and antagonistic. Due to technical limitations and sample acquisition difficulties, the pharmacokinetic study on a single active component has become a great challenge. On top of that, synergistic nature of CHM compounds makes the study even more complicated.

There has long been an interest in CHM innovation and pressing demand for drug development in the field of material basis of CHM research. Researchers who hold “Reductionist” opinions worldwide have launched a research campaign focusing on herbal medicinal chemical compositions and their role in CHM efficacy. Some studies have tried to isolate the primary compound that is directly associated with the pharmacodynamics of a CHM prescription through direct separation analysis of herbal chemical compositions [10, 11]. This approach helps isolate the principal components, partly identify their role played in the overall efficacy, and also describe the consistency of them. But there are difficulties in tracing processes, combined effects of multiple components introduce complexities and affect accuracy of the relevancy description in medicinal efficacy. Thus, it is almost impossible to study the synergistic medicinal value of multiple components. The analysis cannot reflect the influence of synergy by principal component and other components and also the composite effect of entire herbal compound. Other researchers premise the objective to find and identify the common components of serum samples and herbal materials. They set studies to focus on the common components presence in serum sample and herbs, evaluate the pharmacological and pharmacodynamic activities, and finally interpret the correlations of the common components with the overall CHM pharmacodynamics [12]. Although this approach demonstrates the synergistic effects of different CHM components, it is incapable of interpreting the extent contribution of a single component to whole efficacy.

## 4. Advantages of Targeted Depletion of Principal Components

In the interpretation of the material basis of CHM, one approach for multicomponent analysis in herbal medicine is to remove one component at a time and eventually identify the target component of interest according to the changes inefficacy. This methodology is derived from the philosophy of reductionism. It highlights the individual component and elicits its pharmacological effect. However, such approach may not be the best option in analysing CHM on the material basis due to the following reasons. (1) Reductionistic thinking is contradictory to the basic philosophy of Chinese medicine, which emphasizes the “on a whole” approach to every matter. (2) The research object is centred on the effect and mechanism of one single component, which can result in a loss of focus of the overall performance of a CHM mixture. (3) The reduction process of ridding one compound at a time eliminates any synergistic or antagonistic activities between compounds and is not suitable for studies that desire multicomponent interactions. Therefore, the steps of “track” then “separation” are not fully applicable to the material basis of CHM.

The goal of pharmacodynamic analysis of whole compound system of CHM material-based research is difficult to realize in the near future. And the approach to track and analyse single active component still possesses a certain degree of technical difficulties and the results obtained are still questionable. New strategies and methodologies are needed. Protein specific immunodepletion assay may be a valuable solution to the technical obstacles mentioned above.

The basic principle of immunodepletion assay is to utilize antigen-antibody reaction to remove specific proteins in a complex CHM mixture. The objective is to establish and study the correlation between one specific protein and the overall efficacy of a CHM prescription. Comparing the pharmacological properties of a CHM mixture before and after protein deletion can then reveal the specific function of that particular protein. The advantage of adopting this method is the specific nature of antibody, which can selectively target the protein of interest and eliminate all other undesirable factors. The method not only discloses the pharmacodynamics and pharmacokinetics of a single active component but also reveals properties of other untargeted components and their synergistic behaviors. This method allows us to analyse the target component within the herbs and can accurately describe the degree of contribution of the target component to the overall efficacy without destroying the relations of other components.

After the removal of a target protein in a herbal extract, the protein can then be reintroduced into the solution at variable concentration. And a quantity-efficacy (QE) correlation of that protein can then be generated, which demonstrates that the variation in concentration of a principal component can affect the overall therapeutic performance of a CHM preparation. Obtaining the QE correlation of the principal component can provide valuable support for new drug development. Mixing different monoclonal antibodies to create a cocktail can remove several different target proteins at one time. And through orthogonal design and titration method, the synergistic relationships between different active components can then be revealed. Illustrating such synergistic interactions between CHM compounds can provide better scientific information for future CHM formulation and compatibility studies. The immunodepletion of specific CHM proteins allows the analysis of a single component independent of others. It can also be used to study the combined effects of certain different components or all the components altogether and other various related issues. It is a more reasonable way to carry out future CHM researches.

Monoclonal antibody is currently one of the most important research tools in life science research. And monoclonal antibody against CHM compound, if can be widely accepted, will be a very well-prospected research application. CHM researchers are now learning antibody technologies to study the complexities of herbal medicine. In 2005, Zhang et al. [13] reported a work on SiNi-powder. They utilised antiglycyrrhizin polyclonal antibodies to selectively deplete glycyrrhizin and determine its role in contact hypersensitivity in mice. They proposed the idea that a better way to identify the compound of interest on a molecular basis is to selectively remove certain components. In 2006, Chen et al. [14] also used immunoaffinity chromatography to efficiently remove Naringin for research, proving that the method of high specificity removal of Naringin did not influence other components and is worth promoting. A research article in 2011 described the changes in nitric oxide (NO) and nitric oxide synthase (iNOS protein) levels from a liquorice-specific deletion and glycyrrhizin addition experiment. The studies provided a clear efficacy correlation between glycyrrhizin and liquorice and exhibited the unique technical advantage of utilizing protein specific knockout technique [15]. These exploratory efforts have provided new research ideas on CHM components. The adaptation of protein specific deletion technology using monoclonal antibodies can minimize the effect of other components and focus solely on the target component's identification and pharmacological property determination. This new method will have good prospects in the material-based CHM research and in CHM pharmacological evaluation.

## 5. Monoclonal Antibody in Components Depletion

There is no essential difference between the path of making monoclonal antibodies of TCM components and other small molecules. Scholars around the globe have established several hapten synthesizing methods, such as the carbodiimide method [16] and the active-ester method [17]. These methodologies are mature and can provide valuable technical support for CHM hapten synthesis. With improvements in the synthesis of monoclonal antibody, researches in the small molecule immunoassay depending on the use of monoclonal or polyclonal antibodies have gotten a boost as well. Preliminary applications of these immunoassays include pesticide detection in food, rapid drug testing, and environmental monitoring [18–21]. Some research has even demonstrated a very low hapten detection level reaching femtomolar [22].

In recent years, a variety of monoclonal antibodies against active components in herbal medicine have been successfully prepared against liquorice acid [23], paeoniflorin [24], aconitine [25], ginsenosides Rg1 [26], aristolochic acid [27], berberine [28], saikosaponin [29], and so forth. The emergence of all these different antibodies has promoted development in the related research field, for example, CHM quality assessments, serum CHM concentration testings [30, 31], integrating western blot and immunoassays in the analysis of CHM chemical composition, identification of tissue localization [26]. In 2011, Current Drug Discovery Technologies, an internationally renowned journal, published a series of reviews and research articles focusing on monoclonal antibodies [32–34]. This can be a good indication that monoclonal antibody-based small molecule drug discovery technology has gotten more and more attention and become the new drug discovery platform in the field of metabolic mechanism research.

In contrast, limited by outdated research strategies, the usage of specific protein immunodepletion in the field of CHM basic research has not truly begun. It is also due to the lack of high titer monoclonal antibodies and lack of suitable instruments. Another reason for the setback is that more than 90% of the small molecules (MW < 2500) isolated from CHM are haptens, which are incapable of direct stimulation of antibody production in vivo. Another reason is that there are compounds that have similar structures with the active components in a CHM mixture (e.g., isomers) and may cause undesired cross-reaction with the monoclonal antibody, which further complicates the process of polyclonal antibody preparation and antigen synthesis and clone selection. Furthermore, the structures of the active components in CHM are diverse and can create obstacles during the coupling process with macromolecules. Therefore, preparation of high potent monoclonal antibodies for small CHM molecules and establishing a CHM small molecule monoclonal antibody library are the foundations for promoting protein specific immunodepletion technology.

## 6. Research Example

*Pueraria *root (*Radix puerariae*) is a sweet, acrid, cool, and nontoxic herbal remedy. It benefits the spleen and stomach. It is commonly used in the treatment for diarrhoea, fever, thirst, exogenous fever, headache, neck and shoulder pain, chest pain, measles, and so forth. Clinical application of* R*.* puerariae* is widespread in both classic and modern Chinese medicine prescriptions, such as GuiZhiJiaGeGen decoction, GeGenJiaBanXia decoction, GeGenHuanQinHuangLian decoction, Puerarin injection, GanMao QingRe granules, XinKeShu tablets, YuFengNingXin Tablets, TianBaoNingYinXing preparations, and XiaoKe capsules.

*Radix puerariae* contains more than 30 types of flavonoid and isoflavonoid compounds, including daidzein and puerarin. It also contains triterpenoids, steroids, coumarin, GE phenolic glycosides, amino acids, and starch [35]. Puerarin is a type of isoflavone found in* Pueraria*. A typical* Pueraria* usually contains from 3% to 5% of puerarin, which is the primary therapeutic component of* Pueraria* [36]. Testing for the presence of puerarin is a commonly accepted practice to determine the quality of a* Pueraria *root and also as a QC standard for any medications that use* Pueraria.*

Puerarin's pharmacological activity has been a primary research focus in the field of medicine. Literatures suggest that the pharmacological action of* Pueraria* is associated with its antisympathetic, calcium antagonistic, and extensive *β* receptor blocking effects which result in vasodilation, lowering blood sugar and lipoproteins, and lowering blood pressure and subsequently protects the heart, liver, and kidneys [37–40]. The medicinal value of purarin is well established, but questions such as “how is the variation of puerarin concentration in different kinds of* Pueraria* root affecting* Pueraria's *efficacy?” and “should puerarin be taken as the QC of any* Pueraria's* preparations?” need to be explicitly delineated in order to guide the real-world usage to* Pueraria*.

Series of research in the production of monoclonal antibody against the active CHM component were launched since 2008. The study involves the synthesis of artificial antigens by conjugating CHM micromolecules with macromolecules, such as bovine serum albumin. The conjugate obtained was then used to immunize lab animals and generate corresponding cell fusions, which were then screened for positive clones. Researchers have successfully generated monoclonal antibodies against the hormone geniposide, Radix puerariae-baicalin, berberine, and many other CHM components. Mastering and widely adopting monoclonal antibody technology have made significant advancements in the CHM industry. Geniposide and puerarin immunoassay kits were introduced, allowing the completion of study in allergens in Qingkailing injections. The immunoassay chip used in the study of allergen, chlorogenic acid, in QingKaiLing Injection resulted in a number of patents [41–47], and the study also received recognition of its educational value in 2010.

## 7. Concluding Remarks

By using puerarin monoclonal antibody and immunoaffinity chromatography technology, the puerarin was deleted from* Pueraria* water extract. The puerarin immunoaffinity column prepared had strong specificity to puerarin present in the water extract and was able to delete the puerarin without affecting the remaining components in the solution. The bound puerarin was then eluted and a high purity sample of puerarin was obtained. These tools allow researchers to have a clear understanding on how to conduct a more in-depth study in CHM research.* Radix puerariae* was used here as an example for demonstrating monoclonal antibody technology using mAb deletion. There are animal and cellular models created for the purpose of studying the classic and modern medicinal property of* Pueraria* root, which can be used to collect pharmacological data of* Pueraria* under many different experimental criteria. By comparing the pharmacological properties and pharmacodynamics of* Pueraria *extract before and after undergoing puerarin immunodepletion, the main contribution of puerarin to the overall medicinal property of* Pueraria *can then be easily evaluated, in vivo and in vitro, static and dynamic. As a result, puerarin can then be better utilized in QC, compatibility studies and pharmacokinetic studies of* Pueraria* roots, which expands the usage of* Pueraria* roots in clinical medicine in the future. And also as a result, the experimental foundation for any future CHM studies using target specific deletion techniques was established. However, the methodology introduced in this paper still has its limitations, for instance (1) it is not suitable for identifying unknown CHM components and (2) it is also incapable of complete separation of compounds that have already interacted with others. Taking everything into account, the new research strategy still holds many advantages and has proven to provide more effective and convenient ways of pursuing CHM research and help the modernisation of Chinese medicine.
